# Impact of retinal traction induced by epiretinal membrane on aniseikonia

**DOI:** 10.1038/s41598-024-72048-0

**Published:** 2024-10-23

**Authors:** Masayuki Hirano, Shun Minakawa, Yuta Imamura, Naoko Yamamoto

**Affiliations:** Japanese Red Cross Society Himeji Hospital, 1-12-1 Shimoteno, Himeji, Hyogo Japan

**Keywords:** Aniseikonia, Retina, Optical coherence tomography, Epiretinal membrane, Retinal traction, Medical research, Optics and photonics

## Abstract

We investigated the effect of retinal traction caused by epiretinal membranes (ERMs) on aniseikonia and retinal microstructures in 81 unilateral ERMs. Retinal traction was quantified by measuring the maximum depth of the retinal fold (MDRF) using en face optical coherence tomography (OCT) images. Measurements included the mean inner nuclear layer (INL), outer plexiform layer (OPL), outer nuclear layer (ONL), central retinal thickness (CRT), and interocular ratios of the foveal avascular zone (FAZ) area (FAZ ratio). Significant correlations were found between the preoperative MDRF and preoperative aniseikonia (*P* < 0.001), INL thickness (*P* < 0.001), CRT (*P* < 0.001), and FAZ ratio (*P* = 0.003). Preoperative aniseikonia was significantly correlated with preoperative INL and OPL-ONL thicknesses (*P* < 0.001 and *P* = 0.020, respectively) and CRT (*P* = 0.003). Multiple regression analysis revealed that preoperative aniseikonia was significantly associated with preoperative MDRF, INL, and OPL-ONL thicknesses (*P* = 0.029, 0.006, and 0.006, respectively). Twenty-nine eyes underwent membrane peeling, resolving all retinal folds 6 months postoperatively. A significant correlation was observed between preoperative MDRF and postoperative aniseikonia (*P* = 0.011). Our findings suggest that retinal traction by ERM is significantly associated with aniseikonia both pre- and postoperatively, alongside other OCT parameters.

## Introduction

The epiretinal membrane (ERM) is an abnormal fibroproliferative tissue that develops on the internal limiting membrane (ILM) of the retina in the macular region and causes various visual dysfunctions^1–3^. Aniseikonia and metamorphopsia are typical visual impairments associated with ERM^4–11^. Aniseikonia, which affects the quality of vision (QOV), presents symptoms in sensitive individuals, with an aniseikonia ranging from 1 to 3%. Aniseikonia of ≥ 3% causes binocular impairment, and that of ≥ 5% completely impairs binocular vision^12,13^. Hence, it is critical to develop treatment strategies to prevent ERM-induced aniseikonia from affecting the QOV.

Visual dysfunction in patients with ERMs is commonly attributed to retinal traction, yet a quantitative method for evaluating the traction on the retina is lacking. The recent advent of swept-source optical coherence tomography (SS-OCT), with its enhanced penetration and scan speed compared to conventional spectral-domain OCT, has made it possible to capture three-dimensional (3D) images of the retinal structure. Utilizing en face images constructed from these 3D scans, the distribution of ERM on the retinal surface and changes in the retinal structure caused by ERM-induced retinal retraction, such as retinal folds, can be visualized at an arbitrary retinal depth from a bird's-eye view. Furthermore, it is generally known that when folds occur in a thin elastic membrane, the maximum amplitude of folds increases with rising compressive stress^14–16^. Therefore, by measuring the depth of retinal folds using en face images, retinal traction force can be quantitatively evaluated. Specifically, studies have reported that the maximum depth of retinal folds within a 3-mm-diameter circle centered on the macula (MDRF) correlates with metamorphopsia in patients with ERM^17–22^. Despite aniseikonia being a primary contributor to the decline in QOV caused by ERM, there are no reports on the relationship between the percentage of aniseikonia and retinal traction. It is, therefore, necessary to elucidate the association between retinal traction and aniseikonia to understand the strength of traction impacting QOV due to aniseikonia and to apply this knowledge to clinical practice.

Secondary changes in retinal structure induced by ERM traction include the thickening of specific retinal layers and a reduction in the foveal avascular zone (FAZ), both correlated with the degree of metamorphopsia and aniseikonia^4–9,23–26^. However, the mechanisms by which ERM causes these changes remain unclear. Given that retinal traction is a major pathology of ERM, it is necessary to clarify the relationship between retinal traction and these retinal alterations.

In this study, we quantified retinal traction in patients with ERMs by measuring the MDRF and examined its relationship with the incidence of aniseikonia. Additionally, we investigated the relationship between MDRF and OCT parameters, such as mean inner nuclear layer (INL) thickness, mean outer plexiform layer (OPL)-outer nuclear layer (ONL) thickness, central retinal thickness (CRT), and intraocular FAZ ratio, all previously reported to be associated with aniseikonia. Furthermore, we investigated the relationship between MDRF and the percentage of postoperative aniseikonia in patients who had undergone surgical ERM resection.

## Results

### Relationship between pre- and postoperative aniseikonia and preoperative MDRF

**Fig. 1:**
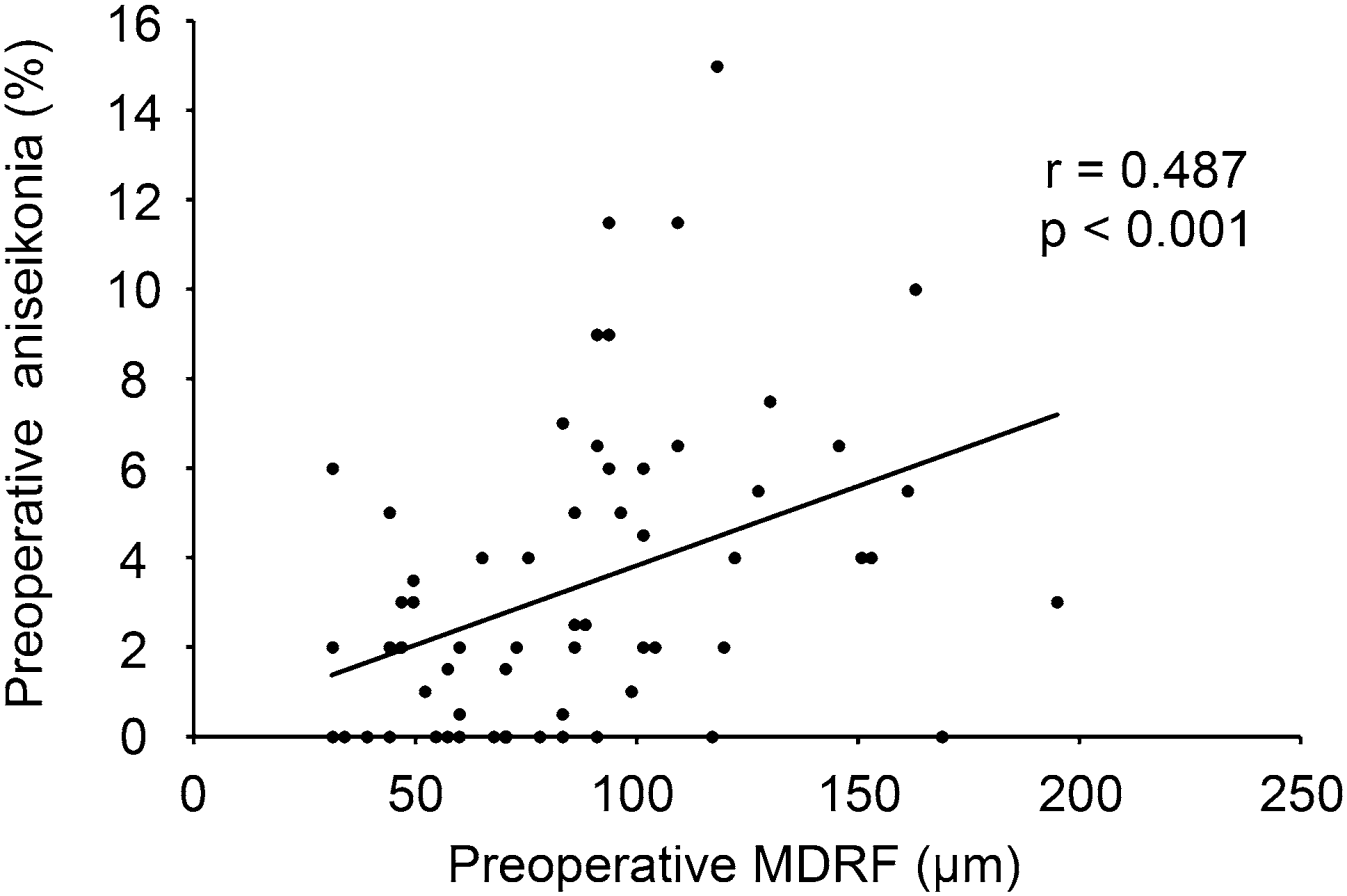
Correlation between the preoperative maximum depth of the retinal fold (MDRF) and mean preoperative aniseikonia.

**Fig. 2:**
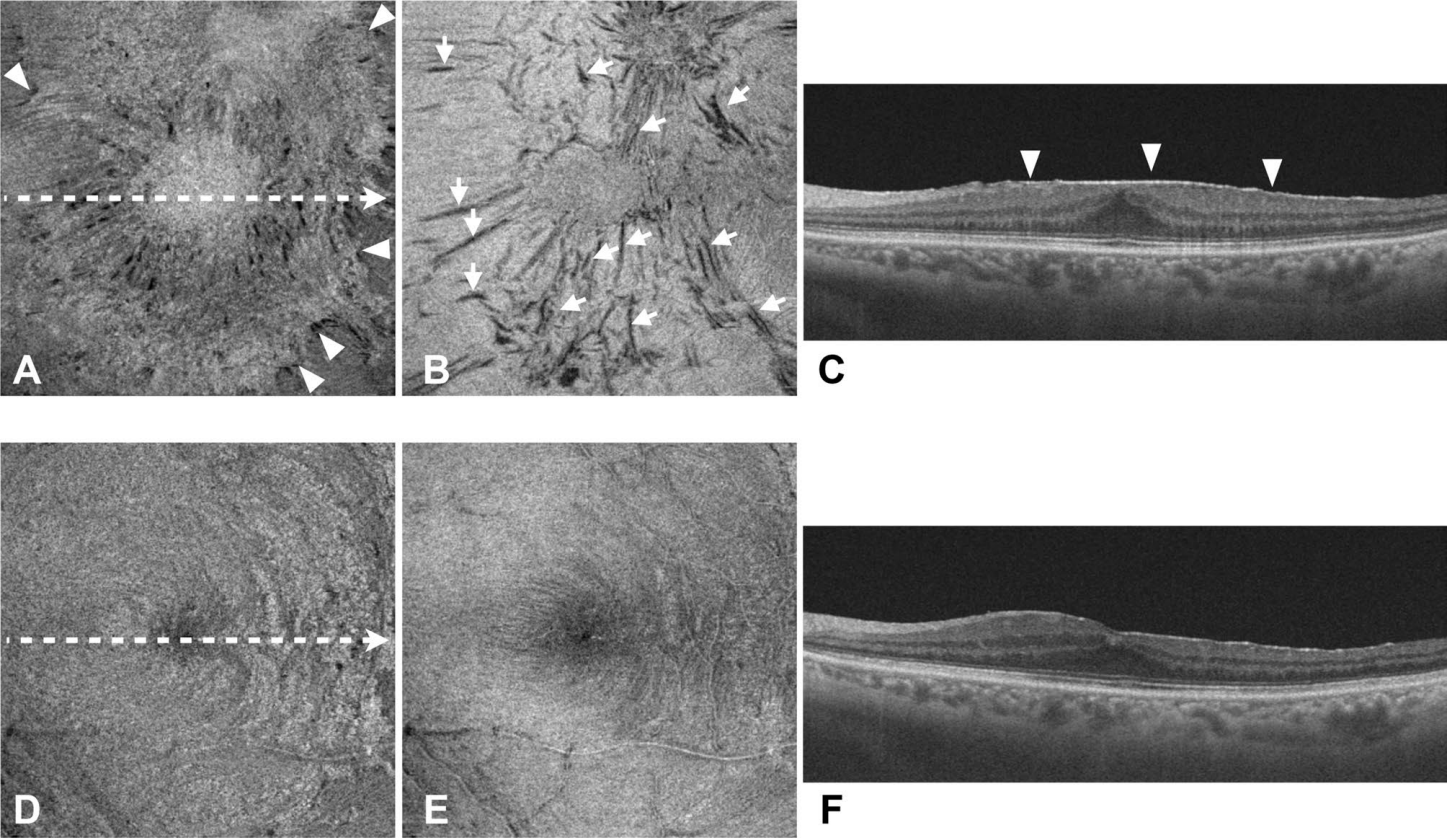
Representative images from a 64-year-old man (**a**–**c**) before and (**d**–**f**) 6 months after epiretinal membrane (ERM) surgery. En face images at the (**a**, **d**) internal limiting membrane (ILM) level and (**b**, **e**) 13.0 μm below the ILM are shown. (**c**, **f**) The white dotted arrows in (**a**) and (**d**) indicate the scan sections of the B-scan images. The white arrowheads in (**a**) and (**c**) indicate ERM. (**a**) ERM in the en face image at the ILM level shows an irregular surface. (**b**) Multiple retinal folds, depicted as black linear structures (white arrows), preoperatively are no longer present in (**e**) the en face image 6 months postoperatively. The maximum depth of retinal folds preoperatively was 62.4 μm. The mean percentage of aniseikonia before and at 6 months after surgery was + 3.0% and + 1.0%, respectively. The best-corrected visual acuity (BCVA) before and at 6 months after surgery was 20/20 and 20/12, respectively.

**Fig. 3:**
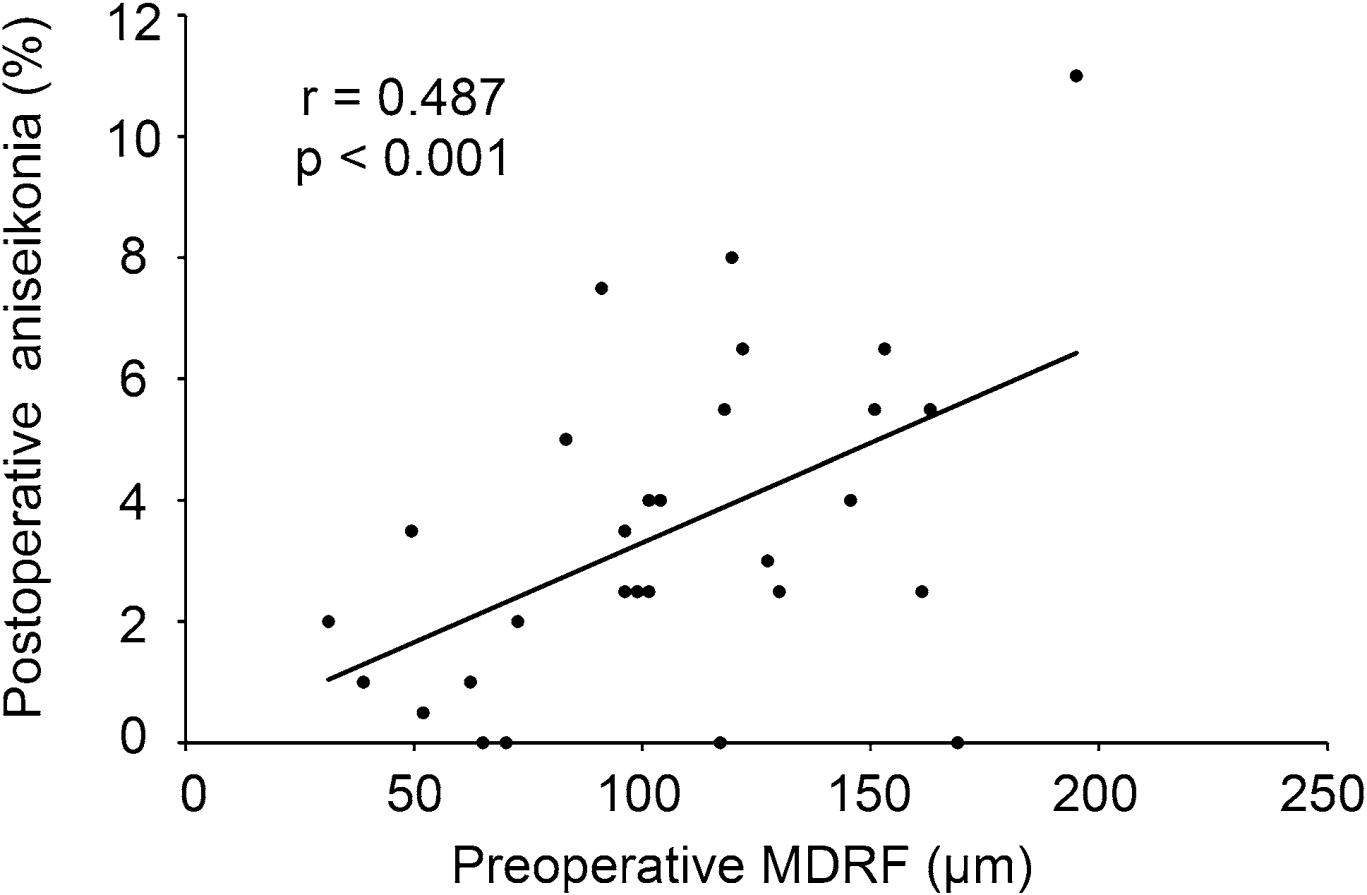
Correlation between preoperative MDRF and the mean percentage of aniseikonia 6 months postoperatively.

We examined the relationship between the preoperative mean aniseikonia and the preoperative MDRF. A significant correlation was observed between the mean percentage of preoperative aniseikonia and preoperative MDRF (y = 0.0417x–0.0757; r = 0.487; *P* < 0.001; Fig. 1). Twenty-nine eyes of 29 patients (15 men and 14 women) with a mean age of 67.5 ± 7.1 years underwent vitrectomy with ERM and ILM peeling, with 22 eyes (75.8%) undergoing simultaneous cataract surgery. No surgical complications occurred during or after surgery in any case. In all cases, retinal folds within a 3-mm-diameter circle centered on the macula were present preoperatively but disappeared by 6 months postoperatively, as illustrated in Fig. 2. The mean MDRF improved from 106.4 ± 41.7 μm preoperatively to 0 μm postoperatively (Table 1). We examined the relationship between the mean percentage of aniseikonia 6 months postoperatively and the preoperative MDRF. The correlation between the mean percentage of aniseikonia at 6 months postoperatively and the preoperative MDRF was also significant (y = 0.0368x–0.5893; r = 0.467; *P* = 0.011; Fig. 3). According to the regression line equations, the preoperative MDRF values corresponding to the threshold at which preoperative and postoperative aniseikonia interfered with daily life (aniseikonia of 3%) were 73.75 μm and 97.53 μm, respectively.

**Table 1:** Comparison of preoperative and 6-month postoperative visual functions and optical coherence tomography findings.

	Baseline	6 months postoperatively	*P-*value
MRDF (μm)	106.4 ± 41.7	0	< 0.001
Mean aniseikonia	3.89 ± 3.33	3.51 ± 2.71	0.508
LogMAR BCVA	0.10 ± 0.22	− 0.06 ± 0.13	< 0.001
INL thickness (μm)	55.2 ± 12.2	50.1 ± 9.30	0.007
OPL-ONL thickness (μm)	101.8 ± 13.6	112.5 ± 12.5	< 0.001
Central retinal thickness (μm)	423.7 ± 79.6	359.8 ± 35.8	< 0.001
FAZ ratio	0.443 ± 0.35	0.393 ± 0.24	0.288

*BCVA* best-corrected visual acuity, *FAZ* foveal avascular zone, *INL* inner nuclear layer, *LogMAR* logarithm of the minimum angle of resolution, *MDRF* maximum depth of retinal folds, *OPL-ONL* outer plexiform layer-outer nuclear layer.

Data are presented as mean ± standard deviation unless otherwise indicated.

### Relationship between preoperative MDRF and preoperative OCT parameters

**Fig. 4:**
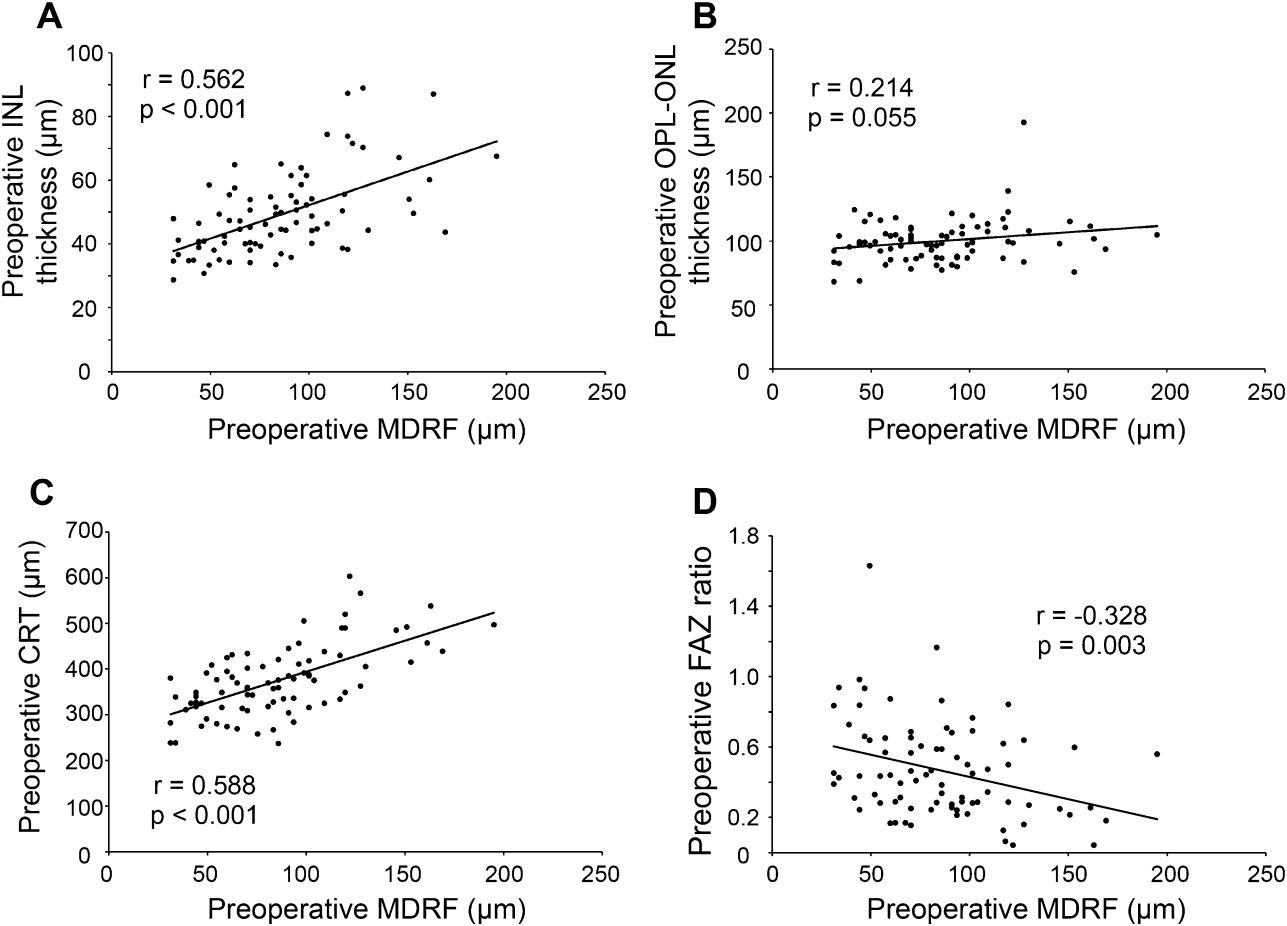
Correlation between preoperative MDRF and optical coherence tomography (OCT) parameters: (**a**) mean preoperative inner nuclear layer (INL) thickness, (**b**) mean preoperative outer plexiform layer (OPL)-outer nuclear layer (ONL) thickness, (**c**) preoperative central retinal thickness (CRT), and (**d**) preoperative foveal avascular zone (FAZ) ratio.

The correlation between preoperative MDRF and OCT parameters associated with aniseikonia, such as the mean INL and OPL-ONL thickness, CRT, and intraocular FAZ ratio, was examined. As shown in Fig. 4, the preoperative MDRF was significantly correlated with the mean preoperative INL thickness (r = 0.562, *P* < 0.001), preoperative CRT (r = 0.588, *P* < 0.001), and preoperative FAZ ratio (r = − 0.328, *P* = 0.003), but not with the mean preoperative OPL-ONL thickness (r = 0.214, *P* = 0.055).

### Relationship between preoperative aniseikonia and preoperative OCT parameters

**Fig. 5:**
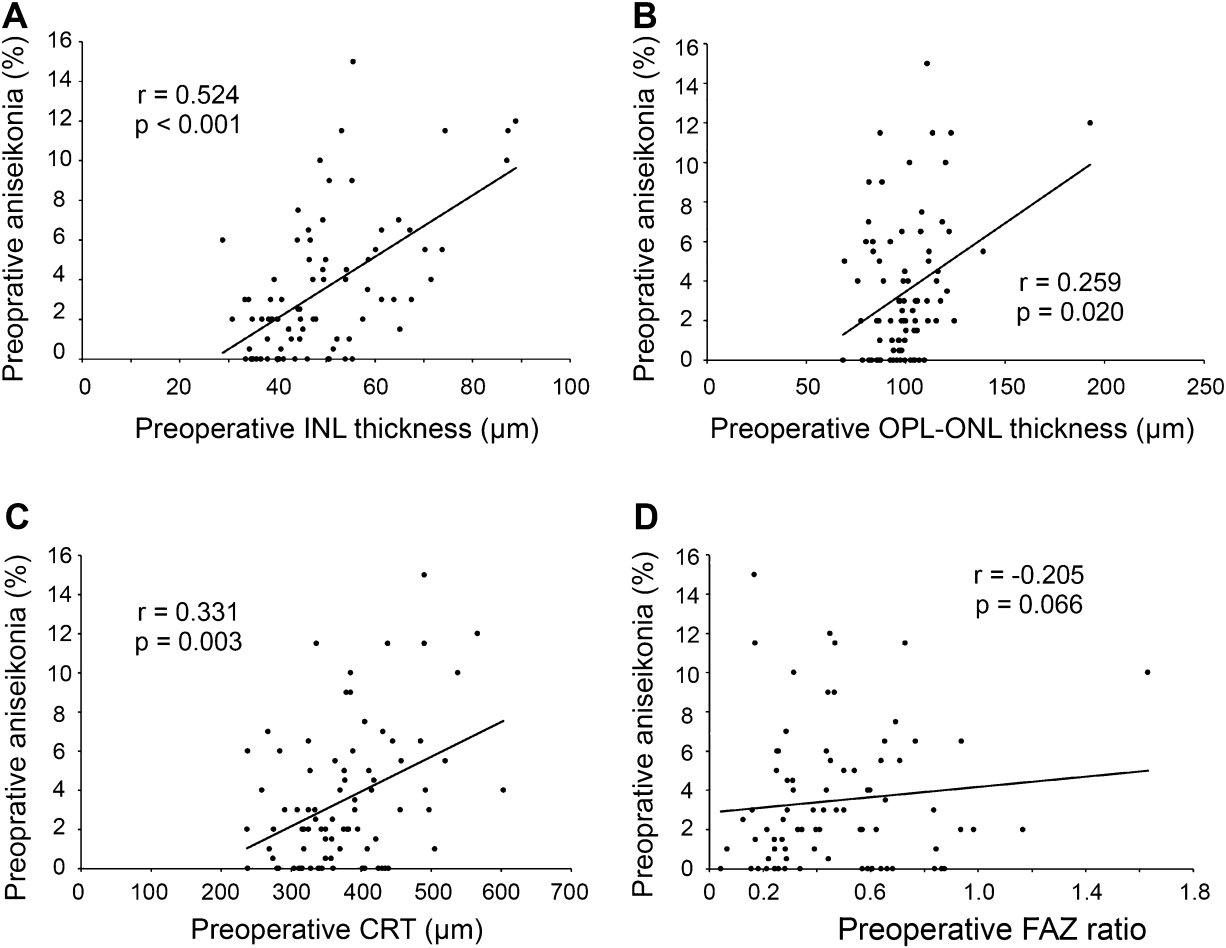
Correlation between the mean percentage of preoperative aniseikonia and preoperative OCT parameters: (**a**) mean preoperative INL thickness, (**b**) mean preoperative OPL-ONL thickness, (**c**) preoperative CRT, and (**d**) preoperative FAZ ratio.

We examined the relationship between the mean percentage of aniseikonia preoperatively and preoperative OCT parameters, such as the mean INL and OPL-ONL thickness, CRT, and intraocular FAZ ratio. As shown in Fig. 5, the mean percentage of aniseikonia preoperatively significantly correlated with the mean preoperative INL and OPL-ONL thicknesses (r = 0.524, *P* < 0.001; r = 0.259, *P* = 0.020; respectively) and preoperative CRT (r = 0.331, *P* = 0.003), but not with the preoperative FAZ ratio (r = -0.328, *P* = 0.066).

### Multiple regression analysis for aniseikonia

Multiple regression analysis was conducted with the mean percentage of aniseikonia preoperatively as the dependent variable and preoperative MDRF, mean preoperative INL and OPL-ONL thicknesses, and preoperative CRT as independent variables. As shown in Table 2, the preoperative MDRF, mean preoperative INL, and OPL-ONL thicknesses were significantly associated with the mean percentage of aniseikonia preoperatively (*P* = 0.029, 0.006, and 0.006, respectively).

**Table 2:** Multiple regression analysis of preoperative aniseikonia.

	β	SE	*P*-value
Preoperative MDRF (μm)	0.271	0.013	0.029
Preoperative INL thickness (μm)	0.457	0.457	0.006
Preoperative OPL-ONL thickness (μm)	0.303	0.303	0.006
Central retinal thickness (μm)	− 0.316	0.009	0.071

*Β* standard regression coefficient, *INL* inner nuclear layer, *MDRF* maximum depth of retinal folds, *OPL-ONL* outer plexiform layer-outer nuclear layer, *SE* standard error.

R^2^ = 0.344.

### Effect of surgical removal of ERM and ILM on visual functions and en face image findings

As shown in Table 1, significant improvements were noted in the best-corrected visual acuity (BCVA), mean INL thickness, and CRT 6 months postoperatively (*P* < 0.001, 0.005, and < 0.001, respectively). However, there was no significant improvement in the mean percentage of aniseikonia (*P* = 0.512). A significant increase in the mean OPL-ONL thickness was observed (*P* < 0.001).

## Discussion

Our current study revealed, for the first time, that both preoperative and postoperative aniseikonia caused by ERM correlate with preoperative MDRF. These results suggest that MDRF measurements may have clinical applications in determining the timing of surgery. Although ERM causes aniseikonia due to retinal traction, mild aniseikonia does not adversely affect daily life. However, aniseikonia causes binocular vision dysfunction in patients with aniseikonia of ≥ 3% and fusion disorder in patients with aniseikonia of ≥ 5%^13^. Therefore, preventing aniseikonia from reaching 3% is crucial in ERM treatment. Using 3% of aniseikonia as the cutoff, the MDRF value corresponding to 3% was calculated to be 73.7 μm based on the relationship between preoperative MDRF and preoperative aniseikonia (y = 0.047x–0.0757). Similarly, the MDRF value corresponding to 3% was calculated from the relationship between preoperative MDRF and postoperative aniseikonia (y = 0.0329x + 0.0159) to be 90.7 μm. In other words, the timing of surgical intervention to remove traction when the MDRF is between 73.7 and 90.7 μm can be considered before the aniseikonia significantly affects the patient's daily life. Preoperative and postoperative MDRF in ERM have also been reported to correlate with metamorphopsia^19,23^. Kanzaki et al. suggested that surgical treatment should be performed when the MDRF is between 69 and 118 μm, using 0.5 as the cutoff for the M-score value at which metamorphopsia affects daily life^19^. This indicates that the timing of surgical treatment before visual dysfunction due to aniseikonia and metamorphopsia caused by ERM interferes with daily life based on MDRF values. The timing of surgical treatment is considered to have the same level of traction for the prevention of visual function due to inequality and prevention of visual function due to metamorphopsia. The results of this study and the Kanzaki et al. study indicate that even in situations where aniseikonia and M-score cannot be measured in symptomatic patients, measurement of MDRF by OCT imaging can now tell whether a degree of traction has occurred that would require surgery.

Additionally to preoperative MDRF, preoperative INL and OPL-ONL thicknesses were significant factors in the multivariate analysis of preoperative aniseikonia. Okamoto et al. reported that INL thickness was a significant factor for aniseikonia due to ERM, whereas OPL-ONL thickness was not a significant factor^7^. In the present study, the mean aniseikonia, mean INL thickness, and mean OPL-ONL thickness in all patients were 3.1 ± 3.8%, 49 ± 13 μm, and 100 ± 17 μm, respectively, compared to 6.2 ± 4.5%, 104 ± 33 μm, and 196 ± 24 μm, respectively, in Okamoto et al.'s study. The difference may be because the study by Okamoto et al. was limited to ERM cases that had undergone surgery, whereas the present study included mild cases for which surgery was not indicated. Thus, in addition to MDRF and INL thicknesses, OPL-ONL thickness may also influence aniseikonia in patients with mild ERM.

This study also revealed a relationship between MDRF and OCT parameters, which have previously been reported to be correlated with aniseikonia caused by ERM. Preoperative MDRF significantly correlated with preoperative INL thickness, preoperative CRT, and preoperative FAZ ratio but not with preoperative OPL-ONL thickness. Kanzaki et al. also reported that although INL and OPL-ONL thicknesses correlated with MDRF, OPL-ONL thickness did not correlate with the amount of MDRF progression or thickness change over time^20^. This study revealed that an increase in INL thickness, CRT, and FAZ ratio are secondary structural changes correlating with retinal traction. Furthermore, it appears that OPL-ONL thickness has a weaker relationship with retinal traction compared to other OCT parameters. A more detailed study of the effects of retinal traction on the retinal structure is necessary.

In this study, retinal folds were observed in all 29 eyes that had undergone ERM removal surgery preoperatively but had disappeared in all cases 6 months postoperatively, resulting in the improvement of mean MDRF from 106.4 ± 41.7 μm preoperatively to 0 μm postoperatively. Retinal folds occur due to tangential traction applied to the retina^14–16^. Therefore, their disappearance indicates that the traction applied to the retina was released. However, no significant improvement in aniseikonia was observed postoperatively despite traction removal. Regarding whether aniseikonia improves with surgical intervention in patients with ERM, although there are some reports of significant improvement^23,27^, others report no improvement^7,10^. ERM may cause aniseikonia by altering the distribution of Müller and photoreceptor cells due to traction, with no postoperative improvement attributed to the unchanged cell distribution^7,28^. This appears consistent even in cases where the traction was completely removed by surgical intervention, and all retinal folds disappeared, with improved INL thickness and BCVA, as in the present surgical cases. Detailed studies, including changes in distribution at the cellular level, are needed to examine the effects of ERM traction and the surgical removal of traction on aniseikonia.

The limitations of this study include its retrospective design, small sample size, and relatively short follow-up period. Additionally, factors other than MDRF, such as retinal fold parameters (i.e., number, distribution pattern, and duration of folds), ERM components, and age-related retinal characteristics, may contribute to visual impairment in ERM. Therefore, further investigations are warranted. Retinal traction quantified by MDRF correlates with aniseikonia, and surgery to remove retinal traction before MDRF values reach 73.7 μm and 90.7 μm may prevent QOV reduction due to aniseikonia.

## Methods

### Study design and ethical considerations

This was a retrospective, consecutive observational study. All investigative procedures adhered to the principles outlined in the tenets of the Declaration of Helsinki. The study was approved by the Ethics Committee of the Himeji Red Cross Hospital, Hyogo, Japan (approval no: 2022-23). Informed consent was obtained using an opt-out procedure.

### Subjects

We retrospectively reviewed the charts of a consecutive series of 81 eyes of 81 patients with unilateral idiopathic ERM who visited the Himeji Red Cross Hospital between February 1, 2019 and January 31, 2023. Patients with a history of other retinal diseases, such as age-related macular degeneration, diabetic retinopathy, retinal vein occlusion, uveitis, and anisometropia > 1 dpt, as well as those who had undergone vitreoretinal surgery, were excluded.

### Ophthalmic examinations

All patients underwent comprehensive ophthalmologic examinations before and 6 months after surgery, including BCVA testing with refraction using a 5-m Landolt C acuity chart, indirect and contact lens slit-lamp biomicroscopy, and SS-OCT (DRI OCT-1 Triton; Topcon Corporation, Tokyo, Japan).

### Quantification of aniseikonia

The New Aniseikonia Test (NAT; Handaya, Tokyo, Japan) was utilized to quantify the severity of aniseikonia. This test consists of a book and spectacles and measures aniseikonia by separating binocular vision with red and green filters. Each eye perceives a half-moon printed on a book page. Two half-moons of different sizes in each pair were arranged in series, with the difference varying in increments of 1%. The subjects wore red-green spectacles and viewed the plates to allow the right eye to see one of the half-moons in each pair and the left eye to see the other half-moon. The participants indicated the pair in which the two half-moons appeared to be of equal size. The actual size difference between the half-moons in the pair represented the percentage of the subject's aniseikonia. The NAT target size was 4 cm (visual field angle, 5.7°), and measurements ranging from 1 to 24% were possible. Measurements were done at approximately 40 cm along both the vertical and horizontal meridians, and their mean values were used for data analysis.

### SS-OCT and en face imaging

SS-OCT images were captured in both B-scan and 3D modes (3 × 3-mm area consisting of 320 × 320 A-scans and 6 × 6-mm area consisting of 512 × 512 A-scans); image analysis software, IMAGEnet6, Version 1.22 (Topcon Corporation, Tokyo, Japan), was used for en face and OCT angiography (OCTA) image construction. Based on the retinal layer boundary information, IMAGEnet6 aligned the 3D-OCT volume scan data along a specific retinal layer boundary, generating en face and OCTA images at an arbitrary depth. Additionally, CRT measurements were obtained at the fovea within a 1-mm-diameter circle using the built-in calculation system of the SS-OCT. The quality of OCT images was automatically evaluated by the Topcon image quality factor, with a score ranging from 0 to 100. Low-quality OCT images (image quality factor < 60 or segmentation error) were excluded.

### Measurement of MDRF

**Fig. 6:**
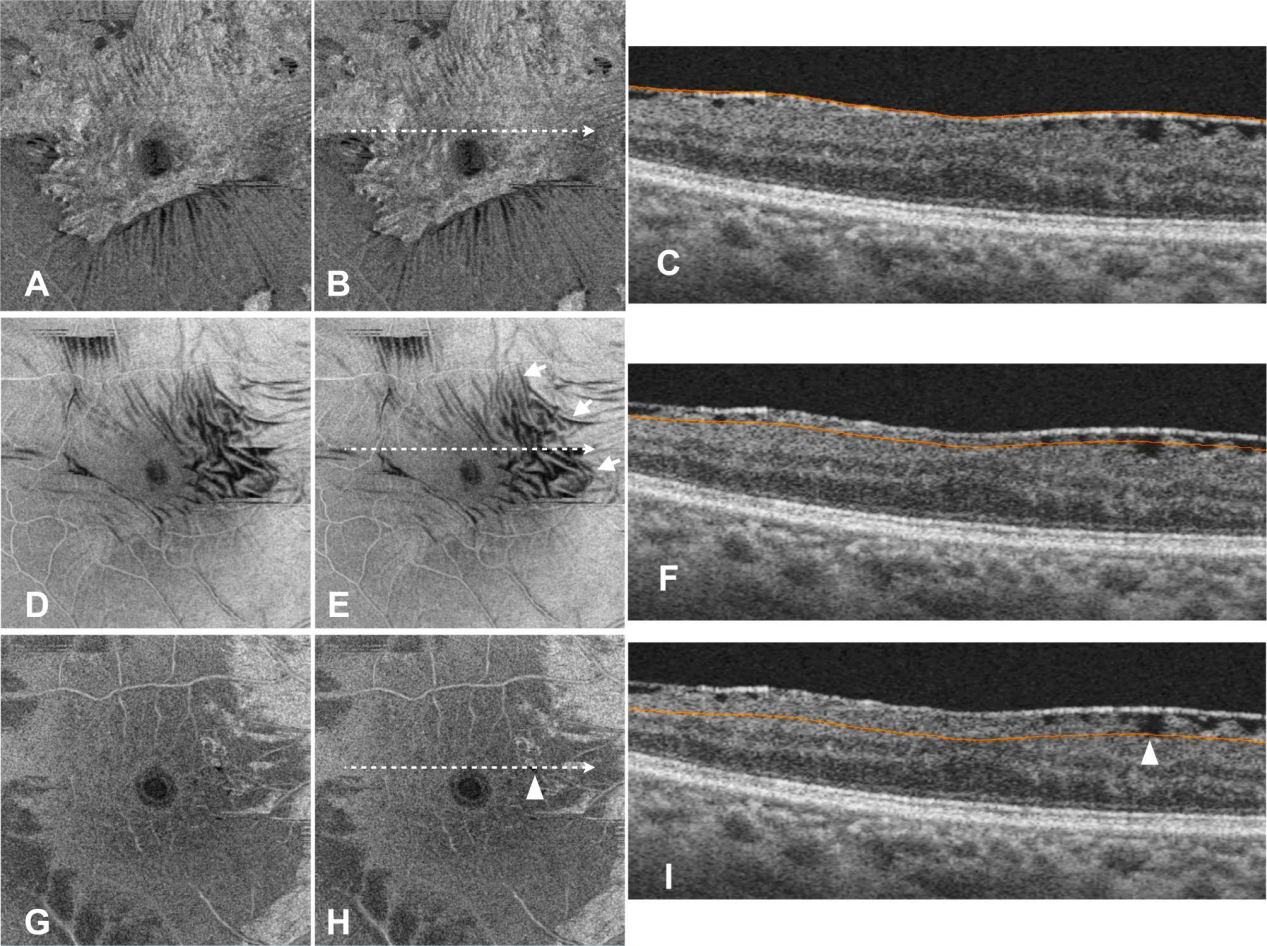
Representative en face images demonstrating the method used to detect the MDRF. En face images (**a**, **b**) at the ILM level, (**d**, **e**) 20.0 μm below the ILM, and (**g**, **h**) 96.2 μm below the ILM, as well as (**c**, **f**, **i**) corresponding B-scan OCT images are shown. (**b**, **e**, **h**) The dotted arrow indicates the location of the B-scan sections. (**c, f, i**) The orange lines in (**c**), (**f**), and (**i**) indicate the slabs at which the en face images of (**a**) and (**b**), (**d**) and (**e**), and (**g**) and (**h**) are made. (**a**, **b**) En face image at the ILM level shows an ERM (indicated by a white arrowhead). (**d**, **e**) En face image at 20.0 μm below the ILM level shows multiple black lines corresponding to retinal folds (white arrows). (**h**) The white arrowhead shows the only retinal fold observed in this slab, indicating the MDRF in the parafoveal area. This retinal fold disappeared in the en face images constructed at a deeper level than this image. Therefore, the MDRF in this case was 96.2 μm. (**i**) The white arrowhead points to the deepest retinal fold, with the orange line passing through its deepest part.

MDRF was measured within a 3-mm-diameter circle centered at the fovea as previously described^17–19^. The 3D OCT volume scan data were flattened at the level of the ILM to visualize the black lines corresponding to the retinal folds due to retinal traction by ERM on the en face image below the ILM level. We then measured the slab depth of the en face image, in which the black lines corresponding to the deepest retinal folds disappeared within the parafoveal area (Fig. 6).

### Measurement of the FAZ Area and the interocular ratios of the FAZ area

Superficial capillary plexus (SCP) en face images from 3 × 3-mm OCTA images were used to measure the FAZ area in both eyes with the ERM and fellow eyes. The en face images of the SCP were constructed from the data 2.6 μm below the ILM and 15.6 μm below the inner plexiform layer. FAZ contour in the SCP was manually traced, and IMAGEnet6 automatically computed the surface area within the drawing-in contour. The interocular ratio of the FAZ area in the ERM eyes to that in the fellow eyes was calculated (Supplementary Fig. S1).

### Measurement of the mean INL and ONL-OPL thicknesses

B-scan OCT images of the vertical and horizontal cross-sections through the fovea were used to measure mean INL and ONL-OPL thicknesses. Measurements were obtained from points located 500 μm and 1000 μm away from the fovea in the superior, inferior, nasal, and temporal regions (1 point at 500 μm and 1 at 1000 μm in each of the four regions; total 8 points; Supplementary Fig. S2). The average of the eight values was used for statistical analysis.

### Surgical procedure

Indications for ERM surgery included decreased visual acuity (< 20/20) or complaints of metamorphopsia or aniseikonia. All patients provided written informed consent preoperatively after the risks and benefits of all surgical procedures were explained to them. In all eyes, triamcinolone acetonide was used intraoperatively to facilitate the visualization of the vitreous and posterior hyaloid. After core vitrectomy using a 27-gauge microincision vitrectomy system (Constellation; Alcon Laboratories, Inc., Fort Worth, Texas, USA), the ERM and ILM were removed using end-gripping forceps. ILM peeling was performed after staining with 0.25 mg/mL brilliant blue G solution (Coomassie BBG 250; Sigma-Aldrich, St. Louis, Missouri, USA). The area of ILM peeling was at least as large as a 3-mm-diameter circle centered on the macula. Cataract extraction with posterior chamber intraocular lens implantation was performed before pars plana vitrectomy in all cataract cases. All the surgeries were performed by a single surgeon (M. H.).

### Statistical analysis

All data were expressed as the mean ± standard deviation. BCVAs were recorded as decimal values and converted to the logarithm of the minimum angle of resolution (logMAR) units for statistical analysis. Visual acuity results were presented as logMAR units and Snellen visual acuities. Statistical analyses were conducted using SPSS, version 24.0.0.0 (IBM Corporation, Armonk, New York, USA). Spearman rank correlation tests were used to assess relationships between the mean percentage of aniseikonia and MDRF, mean INL and ONL-OPL thicknesses, CRT, and FAZ ratio. The relationships between MDRF and the mean INL and ONL-OPL thicknesses, CRT, and FAZ ratio were also analyzed using Spearman rank correlation tests. Multiple regression analysis was conducted with the mean percentage of aniseikonia preoperatively as the dependent variable, preoperative MDRF, mean preoperative INL and OPL-ONL thicknesses, and preoperative CRT as independent variables. The mean aniseikonia, mean INL and ONL-OPL thicknesses, CRT, and FAZ ratio before and 6 months after surgery were compared using the Wilcoxon signed-rank test. A *P*-value of < 0.05 was considered to be statistically significant.

## Supplementary Information

Supplementary Information 1.

Supplementary Information 2.

Supplementary Information 3.

## Data Availability

Data supporting the findings of this study are available from the corresponding author, M.H., upon reasonable request.
