# Supplementary figures and images for: Impact of retinal traction induced by epiretinal membrane on aniseikonia
Source: Sci Rep. 2024 Oct 23;14:25110. doi: 10.1038/s41598-024-72048-0 (PMC11499936; doi:10.1038/s41598-024-72048-0)

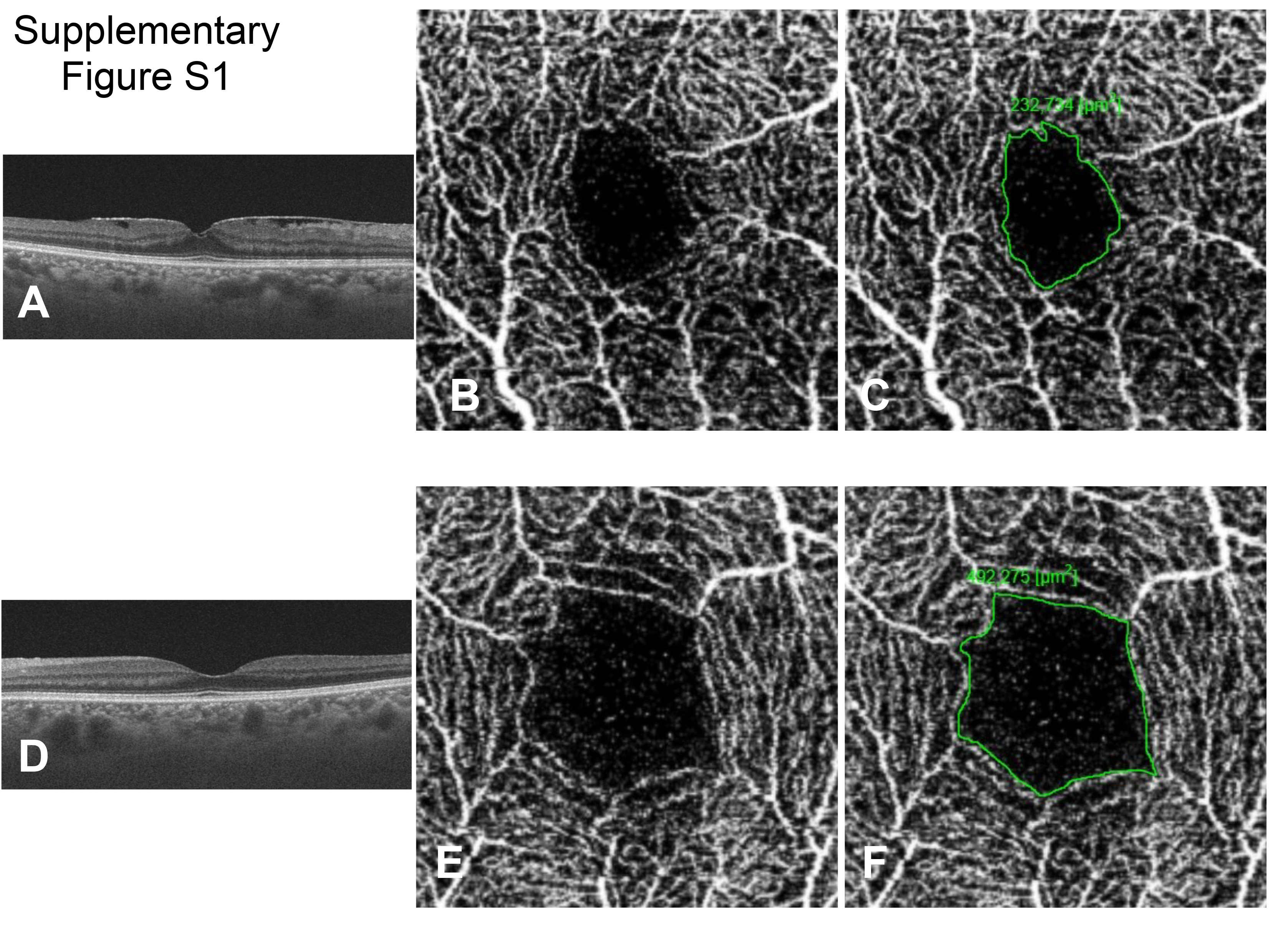

Supplement: Supplementary file 1 — Supplementary Information 1. [file 41598_2024_72048_MOESM1_ESM.tif]

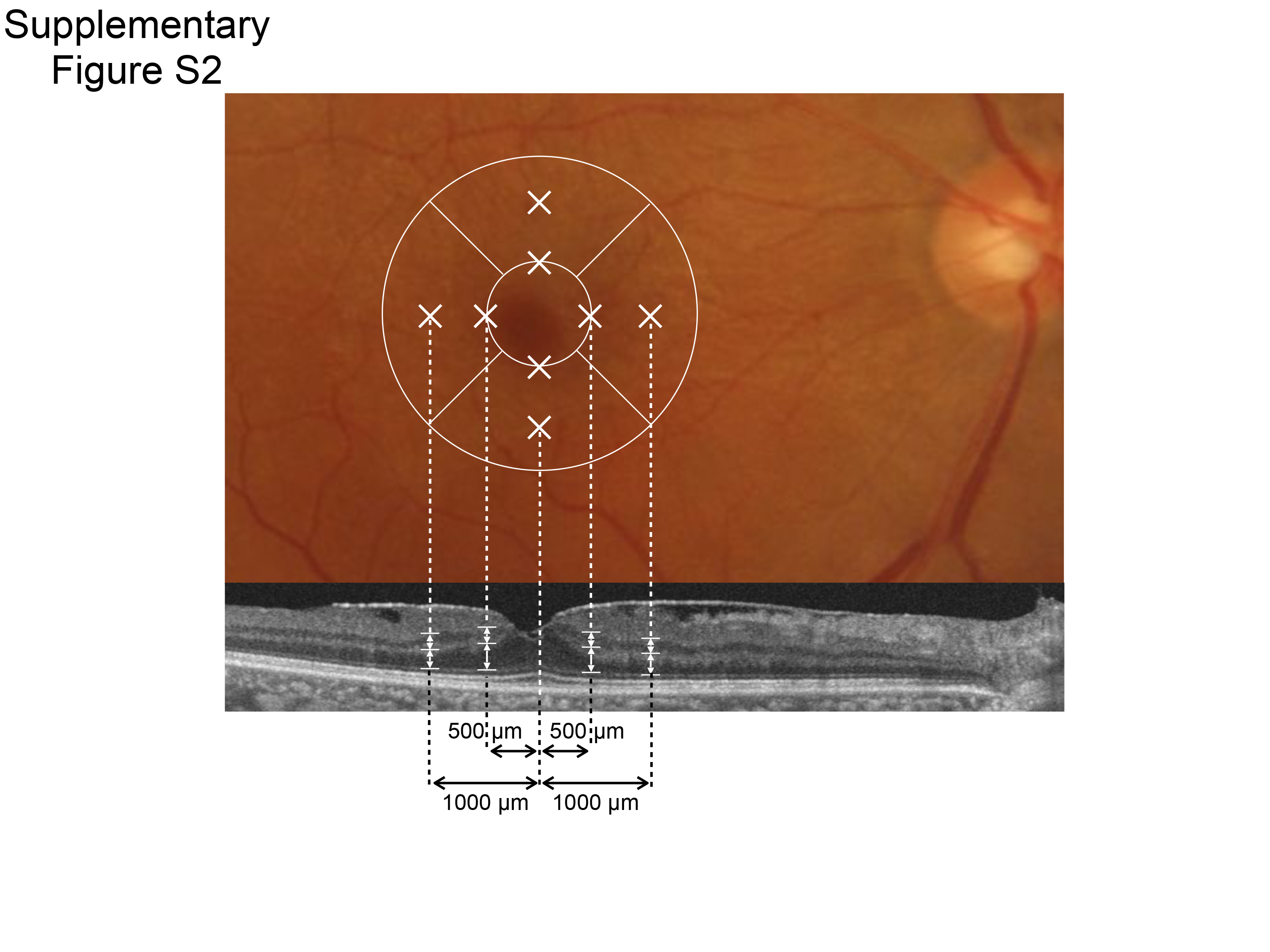

Supplement: Supplementary file 2 — Supplementary Information 2. [file 41598_2024_72048_MOESM2_ESM.tif]
