# Supplementary material for: Impact of retinal traction induced by epiretinal membrane on aniseikonia
Source: Sci Rep. 2024 Oct 23;14:25110. doi: 10.1038/s41598-024-72048-0 (PMC11499936; doi:10.1038/s41598-024-72048-0)
Supplement: Supplementary file 3 — Supplementary Information 3. [file 41598_2024_72048_MOESM3_ESM.docx]

**Supplementary Figure Legends**

**Supplementary Figure S1.**

Representative images illustrating the measurement of the foveal avascular zone (FAZ). (**a**, **d**) B-scan images and (**b**, **c**, **e**, **f**) OCT angiography images from (**a**–**c**) the eye with epiretinal membrane (ERM) and (**d**–**f**) the fellow eye without ERM are shown. (**c**) The FAZ of the eye with ERM was 232.7 μm^2^ (area delineated by the green line). (**d**) The FAZ of the fellow eye was 192.2 μm^2^ (area delineated by the green line). Therefore, the FAZ ratio in this case was calculated as 0.825.

**Supplementary Figure S2.**

Measurements of inner nuclear layer (INL), outer nuclear layer (ONL), and outer plexiform layer (OPL) thicknesses. Measurements were taken at distances of 500 μm and 1000 μm, with the fovea as the center, at eight locations (indicated by white cross marks) in four directions: superior, inferior, nasal, and temporal to the fovea.
